# Algal Oil Rich in n-3 PUFA Alleviates DSS-Induced Colitis via Regulation of Gut Microbiota and Restoration of Intestinal Barrier

**DOI:** 10.3389/fmicb.2020.615404

**Published:** 2020-12-16

**Authors:** Zhenxia Xu, Hu Tang, Fenghong Huang, Zhixian Qiao, Xu Wang, Chen Yang, Qianchun Deng

**Affiliations:** ^1^Oil Crops and Lipids Process Technology National and Local Joint Engineering Laboratory, Oil Crops Research Institute, Chinese Academy of Agricultural Sciences, Wuhan, China; ^2^Institute of Hydrobiology, Chinese Academy of Sciences, Wuhan, China; ^3^College of Animal Science and Technology, Huazhong Agricultural University, Wuhan, China

**Keywords:** algal oil, docosahexaenoic acid, gut microbiota, intestinal barrier, colitis

## Abstract

Algal oil is rich in docosahexaenoic acid (DHA) and has various health benefits against human metabolic disorders and disease. This study aimed to investigate the effects of DHA algal oil on colonic inflammation and intestinal microbiota in dextran sulfate sodium (DSS)-induced colitis mice model. Male C57BL/6 mice was induced colitis by 2.5% DSS and followed by 2 weeks of treatment with algal oil (250 or 500 mg/kg/day). The colonic inflammation was assessed by colon macroscopic damage scores, and the degree of neutrophil infiltration was evaluated by measuring tissue-associated myeloperoxidase (MPO) activity in colonic mucosa. Tight junction proteins in the colonic tissue were measured by real-time PCR and western blot. Moreover, the intestinal microbiota and shot chain fatty acids (SCFAs) were estimated by bioinformatic analysis and GC, respectively. Colonic damage due to DSS treatment was significantly ameliorated by algal oil supplementation. In addition, algal oil significantly inhibited the increases of malondialdehyde (MDA) content, MPO activity, pro-inflammatory cytokines level and tight junction proteins expression in DSS-treated mice. Furthermore, supplementation of algal oil modulated the intestinal microbiota structure in DSS induced colitis mice by increasing the proportion of the *unidentified_S24_7* and decreasing the relative abundance of *unidentified_Ruminococcaceae*, *Clostridium and Roseburia*. On the analysis of SCFAs, the caecal content of acetic acid, propionic acid, isobutyric acid, buturic, and the total SCFAs showed a significant increase in algal oil-administered mice. Together, these results suggested that algal oil rich in DHA inhibited the progress of DSS-induced colitis in mice by modulating the intestinal microbiota and metabolites and repairing the intestinal barrier, which may be applied in the development of therapeutics for intestinal inflammation.

## Introduction

As the recurrent and chronic inflammatory diseases, inflammatory bowel diseases (IBD) have been found to be becoming commonplace around the world (Keethy et al., 2014; Ng et al., 2018). It represents a series of chronic relapsing-remitting gastrointestinal tract disorders characterized by a persistent intestinal inflammation with clinical symptoms of abdominal pain, blood in stool, weight loss, diarrhea, fatigue, or maldigestion (Hanauer and Hommes, 1996; Yu and Rodriguez, 2017). The precise pathogenesis of IBD is not exactly known, however, some researchers agree that IBD is associated with environmental factors, intestinal microbiota disorder, abnormal autoimmune responses, and genetic susceptibility (Danese and Fiocchi, 2006; Italia and Claudio, 2015; Park et al., 2019). To date, the treatment options of IBD are mainly concentrated on conventional therapies, including non-specific immunosuppressive therapies, antibiotics and anti-inflammatory agents, which have low efficacy and may even cause many side effects (Baumgart and Sandborn, 2007; Yamamoto-Furusho, 2018). Thus, developing replaceable therapies and searching for new, safe, and effective nutritional or dietary products are of importance to treatment of IBD.

IBD is closely related to intestinal epithelial barrier dysfunction, abnormal immune response and gut microbiota dysbiosis (Harris and Chang, 2018; Li et al., 2019). Intestinal epithelial barrier function mainly depended on tight junctions (TJs) between adjacent intestinal epithelium. TJs comprise of TJ proteins, primarily Claudin-1, Occludin, and zonula occludens-1 (ZO-1) (Zeisel et al., 2018), and the abnormal expression of TJ proteins increased intestinal permeability and facilitated pathogen invasion, inducing immunity dysfunction and the occurrence of IBD (Deng et al., 2017). In addition, innate and adaptive immune systems and their secreted mediators (cytokines and chemokines) are implicated in inflammation process (Keren and David, 1992). Cytokines exhibit bidirectional regulating effects on immune response as modulators of T cell differentiation into alternative T helper cells and regulatory T cells, and imbalances in cytokine levels contribute to IBD (Aaron et al., 2008). Changes in structure and composition of gut microbiota might occur during the early stage of IBD and precipitate the initiation and progression of the disease, and inflammation itself, in turn, perhaps alter the metabolic conditions to exacerbate gut dysbiosis (Ni et al., 2017). Gut dysbiosis, a microbial imbalance, leads to the increased permeability of the intestinal epithelial mucosa and then attracts lymphocytes and macrophages to release and accumulate inflammatory factors and cytokines, ultimately accelerating inflammation (Nagalingam et al., 2015; Rodiño-Janeiro et al., 2018; Wu et al., 2020).

Recently, the beneficial effects of dietary lipids (especially long chain n-3 polyunsaturated fatty acids) on inflammation have invoked researcher’s great interest (Ma et al., 2019). Several studies have shown that fish oil modulated inflammation responses through specific mechanisms involving alteration of key lipid metabolites, inhibition of the production and release of pro-inflammatory eicosanoids and cytokines, and down-regulation of the activity of related enzymes (Camuesco et al., 2006; Chapkin et al., 2009; Sharma et al., 2018). Previous studies showed that sesame oil exerted the protective effect against 2,4,6-trinitrobenzenesulfonic acid-induced colitis and promoted colon healing by inhibition of inflammation, acidic mucin and fibrosis (Periasamy et al., 2013). In addition, Wu et al. (2020) found that camellia oil ameliorated colitis by inhibiting inflammation and oxidant reaction and promoting the proliferation of beneficial bacteria.

DHA algal oil with a 46.7% DHA content was produced from *Schizochytrium* sp. by the large-scale fermentation technology and has been used in human nutrition as a food ingredient and dietary supplement (Vuorinen et al., 2020). Algal oil has plenty of physiologically active substances, for example DHA, EPA, and DPA which possess anti-inflammatory properties, and algal oil exerts its anti-inflammatory activities via blockade of NF-κB nuclear translocation and inhibition of inflammatory mediator production (Nauroth et al., 2010; Chitranjali et al., 2014). Although it has been demonstrated that algal oil has anti-inflammatory effects in various clinical trials (Calder, 2008), the details how DHA algal oil affects the progression of IBD have not yet been defined. In view of this, the present study was aimed to investigate the effects of algal oil on the structure of intestinal microbiota and intestinal inflammation in the colitis mice induced by dextran sodium sulfate (DSS) and to clarify its mechanism. The results would shield a light on using algal oil to treat IBD and other intestinal dysfunction.

## Materials and Methods

### Chemicals

DHA algal oil was obtained from CABIO Biotechnology Co., Ltd. (Wuhan, China), and the components of algal oil were determined by GC and shown in Supplementary Table S1. It was found that DHA and docosapentenoic acid (DPA) accounted 63.22 ± 0.23% of total fatty acid. DSS with a molecular weight of 36–50 kDa was purchased from MP Biomedicals Co., Ltd (Illkirch, France). The myeloperoxidase (MPO) testing kit was ordered from the Jiancheng Bioengineering Institute of Nanjing (Nanjing, China). The MDA assay kit and BCA protein kit were purchased from Beyotime Biotechnology (Shanghai, China). All enzyme-linked immunosorbent assay (ELISA) kits were ordered from elabscience Biotechnology Co., Ltd (Wuhan, China). Horseradish peroxidase conjugated goat anti-rabbit antibody and primary antibodies raised against Claudin-1, Occludin, and β-actin were provided by Abcam (Cambridgeshire, United Kingdom). And all other chemicals were of reagent grade.

### Animals

Male C57BL/6 mice with 4–5 weeks old and weighed 18–20 g were obtained from Laboratory Animal Center of Huazhong Agricultural University. The mice were kept in specific pathogen-free (SPF) room with 50 ± 5% humidity and a 12 h light/dark cycle and given pellet feed (AIN93M Standard; OpenSource; shown as Supplementary Table S2) and water *ad libitum* throughout the period of experiment. Both animal care and the experimental protocol were in conformity to the Animal Ethics Committee of Experimental Animal Center of Huazhong Agricultural University (SYXK 2017-0044).

### Induction of Colitis and Drug Administration

**FIGURE 1:**
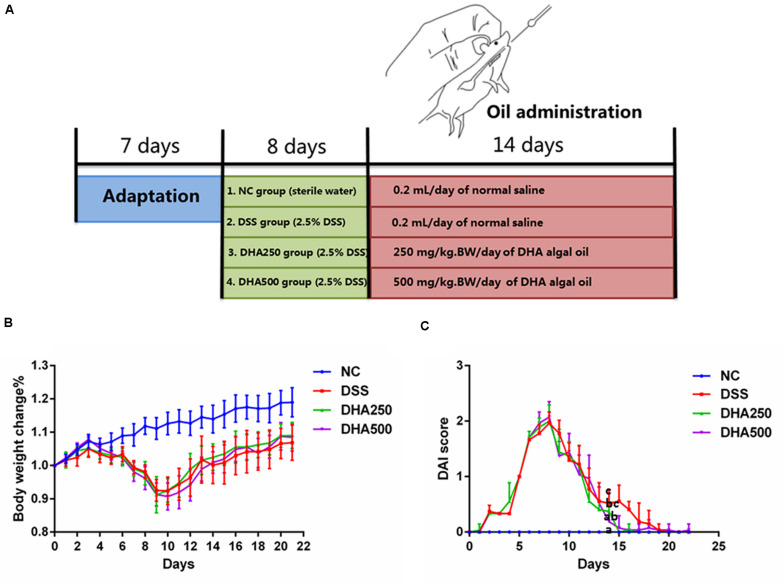
Effects of algal oil on the symptoms of DSS-induced colitis in a mice model. **(A)** Experimental design. **(B)** Body weight change (%). **(C)** Disease activity index (DAI) score. Groups with the same letter are considered to have no significant differences, and groups with different letter are significantly different at *p* < 0.05. Among them, the average data of group with “a” was the minimum while the average data of group with “c” was the maximum. The average data of group with “b” was higher than that of group with “a” and lower than that of group with “c”.

Colitis was induced by giving mice 2.5% DSS drinking water. Algal oil was dissolved in 0.1% flaxseed gum solution for drug administration. The experimental design was schematized in Figure 1A. The mice were randomly assigned to four groups, including nine mice in each: negative control (NC) group, DSS group, DHA250 group and DHA500 group. NC group orally received 0.2 mL/day of the normal saline without induction of colitis. Mice in DSS group were given 2.5% DSS drinking water for 8 days to induce colitis and then received 0.2 mL/day of 0.1% flaxseed gum for 2 weeks. Mice in algal oil treatment groups were orally administered two doses of algal oil (250 and 500 mg/kg⋅Bw/day) for 14 consecutive days after colitis induction.

### Assessment of Colitis Symptoms

DAI-related traits including body weight, stool consistency, and hematochezia were observed and recorded every day, and DAI score was the average of the scoring of weight loss, stool consistency, and hematochezia (Zhao et al., 2017). 0.5 cm distal colon tissues were cut and fixed in 4% paraformaldehyde, and haematoxylin and eosin (HE) stain followed by microscopic observation was performed for histological examination. The MPO assay kit (Jiancheng Bioengineering Institute of Nanjing, Nanjing, China) and MDA assay Kit (Beyotime Biotechnology, Shanghai, China) were used to measure activity of MPO and MDA content in colonic tissues, respectively. Cytokines levels (IL-10, IL-1β, IL-6, and TNF-α) in serum were quantified by ELISA kits (elabscience Biotechnology Co., Ltd, Wuhan, China).

### Reverse Transcription Quantitative Polymerase Chain Reaction (RT-qPCR)

Total RNA of colon tissue sample was extracted by using TRIzol reagent (Invitrogen, Carlsbad, United States). The concentration and quality of RNA were examined by a Nanodrop ND-1000 Spectrophotometer (Thermo Fisher Scientific, Waltham, MA, United States). cDNA was synthesized by cDNA Synthesis SuperMix for qPCR (Yeasen Biotech Co., Ltd, Shanghai, China). And RT-qPCR amplification was conducted using SYBR Green Master Mix (Yeasen Biotech Co., Ltd, Shanghai, China) on Bio-Rad iQ5 Real Time PCR system in triplicates. Relative expression levels of genes were calculated with ΔΔCt method by using reference gene GAPDH to normalization. RT-qPCR primer sequences used were shown in Supplementary Table S3.

### Protein Preparation and Western Blot

Segments of colon were homogenized and lysed in RIPA buffers with PMSF protease inhibitor. Then, the homogenate was centrifuged at 14,000*g* for 5 min at 4°C. Protein concentration of the collected supernatant was quantified by using BCA Protein Assay Kit (Beyotime Biotechnology, Shanghai, China). Loading buffer was added to the supernatant and the mixture was boiled for 10 min at 100°C for the subsequent western blotting analysis. Forty μg of protein was separated on a 10% SDS-PAGE gel by electrophoresis and then transferred to an immobilon PVDF membrane. After blocked with 5% skimmed milk solution in TBST buffer for 2 h at ambient temperature, the membrane was incubated overnight at 4°C with rabbit primary antibody against Claudin-1, Occludin, or β-actin. Subsequent incubation with appropriate respective HRP-conjugated secondary antibody was 1 h at room temperature. And all protein bands were visualized with enhanced chemo-luminescence substrate. The integrated density of each protein band was measured by using ImageJ, and relative protein expression level was calculated by normalizing β-actin level.

### Bioinformatic Analysis

Fecal samples in mice were collected and immediately stored at −80°C for bioinformatical analysis. Total bacterial DNA was extracted from feces using the DNA Kit and the V3–V4 region of 16S rDNA was amplified by using the synthetic universal primers: 338F (5′-ACTCCTACGGGAGGCAGCA-3′) and 806R (5′-GGACTACHVGGGTWTCTAAT-3′). The amplicons were sequenced on the Illumina Miseq platform and sequences with a 97% similarity were grouped into operational taxonomic units (OTUs). The species diversity and richness in gut microbiota were reflected by alpha-diversity indices using QIIME software. Alpha diversity was measured by Chao1, Simpson, Shannon, and Pielou_e indexes. The community richness was measured by Chao1 index, while the microbial evenness was represented with Simpson, Shannon, and Pielou_e indexes. Chao1 index was calculated as ${\text{S}}_{1}={\text{S}}_{\text{obs}}+\frac{{\text{F}}_{1}\,\left({\text{F}}_{1}-1\right)}{2\,{\text{F}}_{2}\,\left({\text{F}}_{2}+1\right)}$, Simpson index was calculated as $\text{D}=1-\sum_{i=1}^{s}\left(P_{i}^{2}\right)$, Shannon index was calculated as H ′$\sum_{i=1}^{s}$(P_*i*_^∗^log_2_P_*i*_) and Pielou_e index was calculated as $\text{J}=\frac{{\text{H}}^{\prime }}{{\text{H}}_{\text{max}}^{\prime }}$, where F_1_ and F_2_ represent the number of Singletons and Doubletons, respectively, s is the total number of identified OUT, P_*i*_ is the proportion of the ith. OTU sequence Taxonomical level and principal co-ordinates analysis (PCoA) were performed by using R software. Linear discriminant analysis (LDA) effect size (LEfSe) was used to identify the specific bacterial taxa at the OTU level.

### Determination of Short-Chain Fatty Acids (SCFAs)

Concentrations of SCFAs (acetic acid, propionic acid, butyric acid, isobutyric acid, valeric acid and isovaleric acid) in cecal samples collected from mice were measured by gas chromatograph-flame ionization detector (GC-FID) with a DB-FFAP capillary column (30 m × 0.53 mm × 0.50 μm, Agilent) on an Agilent 7890N GC system (Zhao et al., 2010). Briefly, cecal samples (50 mg) were resuspended in 250 μL of pure water and acidified with HCl, and then the mixture was centrifuged at 1, 400 rpm for 30 min. 0.5 μL internal standard, 2-ethylbutyric acid solution, was added to the supernatant and the solutions were finally filtered and injected into the column for GC analysis. The carrier gas was N2 and the split ratio was 10:1. The GC temperature program was as follows: begin at 80°C, maintained for 0.5 min, increase to 180°C at the rate of 8°C/min, and maintained for 1 min, followed by 20°C/min to 200°C, and hold at 200°C for 5 min.

### Statistical Analysis

All date are reported as the means ± standard error of the mean (SEM). Difference significance test across subgroups was carried out by using One-way ANOVA for parametric variables and Kruska-Wallis test for non-parametric variables. A *p* < 0.05 was considered statistically significant.

## Results

### Algal Oil Ameliorated the Progress of DSS-Induced Colitis

To determine the anti-inflammatory effects of algal oil, algal oil was orally administered to colitis mice. Body weight, diarrhea and hematochezia were observed and recorded per day throughout the entire experiment. Body weight of the NC group increased gradually over time during the experiment, while DSS-induced colitis mice displayed significant body weight loss by day 7th due to severe colitis and then to increase on day 10th. From Figure 1B, mice treated with algal oil had less body weight loss than DSS mice. As shown in Figure 1C, DAI score of the DSS mice increased on the first day, and it showed significant differences between DSS mice and NC mice. The DAI score of algal oil-treated mice showed an increase on the first day but declined on the 9th day. And a statistically significant difference was also observed between the mice in DHA500 group and DSS mice on the 14th day (*p* < 0.05).

### Algal Oil Alleviated the Symptoms of DSS-Induced Colitis

**FIGURE 2:**
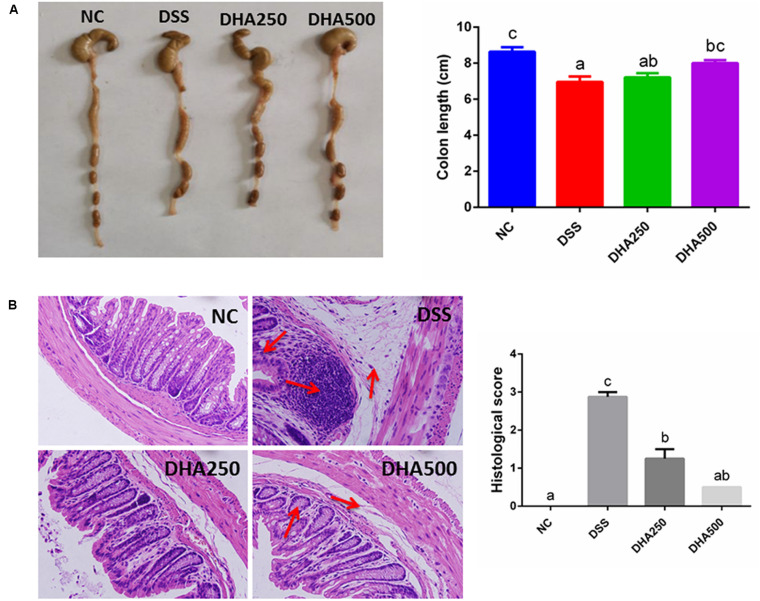
Effects of algal oil on **(A)** colon length, **(B)** hematoxylin and eosin (H&E) staining in mice with DSS-induced colitis. The results are expressed as the mean ± SEM (*n* = 9). Groups with the same letter are considered to have no significant differences, and groups with different letter are significantly different at *p* < 0.05. Among them, the average data of group with “a” was the minimum while the average data of group with “c” was the maximum. The average data of group with “b” was higher than that of group with “a” and lower than that of group with “c”.

Colon length of the mice in DSS group was shorter than that in the NC group (*p* < 0.05) (Figure 2A). Moreover, the colons in the DHA groups, especially in the DHA500 group, were longer than those in the DSS group (*p* < 0.05). Microscopic examination of the colon tissues showed that mice in DSS group exhibited a large field of mucosa erosion and edema, disappearance of superficial epithelial cells and goblet cells, extensive inflammatory-like cell infiltration in lamina propria and submucosa, crypt disruption and loss. Whereas the normal tissue morphology of colon with intact colonic mucosa, neat vill, and healthy crypt structures in the NC group was observed. Mice administered algal oil recovered the mucosal structure compared with DSS-induced colitis mice (Figure 2B). The histological scores were markedly lower in the algal oil-administered group than that in the DSS-induced colitis group (*P* < 0.05).

### Algal Oil Treatment Suppressed the Production of MDA, MPO, and Inflammatory Cytokines in DSS-Induced Colitis

**FIGURE 3:**
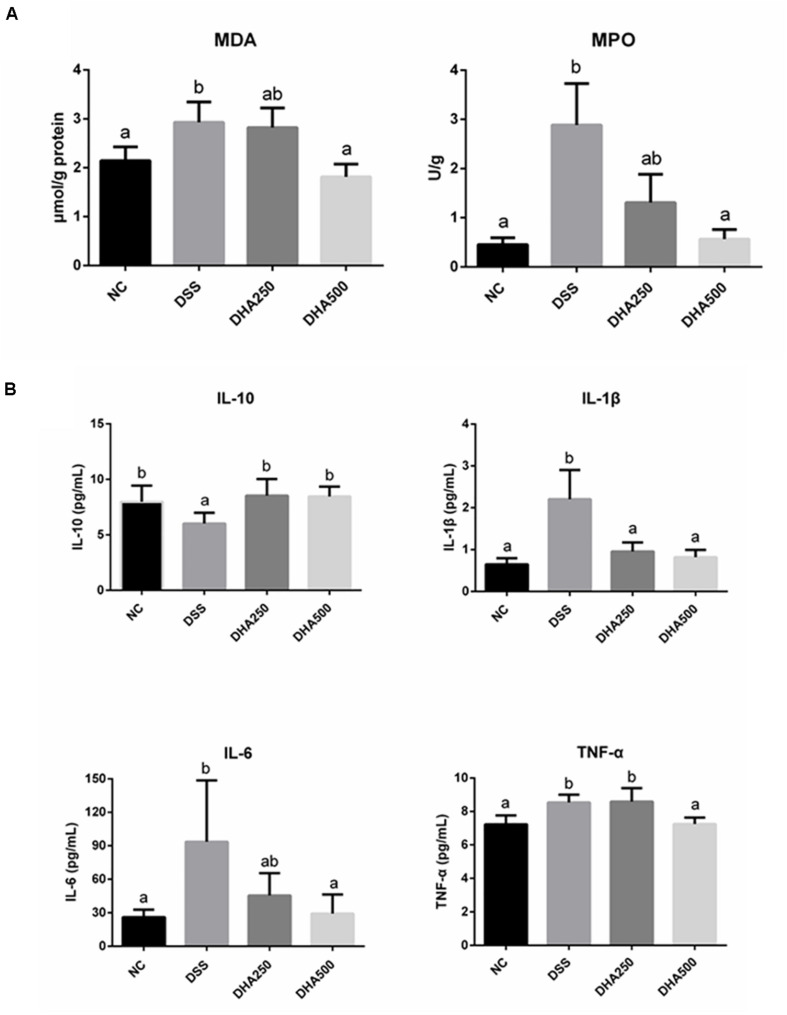
Effects of algal oil on oxidative damage **(A)** and cytokines levels **(B)** of C57BL/6 mice with DSS-induced colitis. The results are expressed as the mean ± SEM (*n* = 9). Groups with the same letter are considered to have no significant differences, and groups with different letter are significantly different at *p* < 0.05. Among them, the average data of group with “a” was the minimum while the average data of group with “b” was the maximum.

The effects of algal oil on the colonic MDA and MPO in the colitis mice were shown in Figure 3A. DSS treatment caused a significant increase in the content of MDA (2.93 ± 0.41 μmol/g) (*p* < 0.05) and MPO (2.89 ± 0.84 U/g) (*p* < 0.01) compared with mice in NC group (2.14 ± 0.28 μmol/g, 0.46 ± 0.13 U/g), respectively. Supplement with algal oil at 500 mg/kg/day reduced MDA to 1.82 ± 0.26 μmol/g (*p* < 0.05) and reduced MPO to 0.57 ± 0.20 U/g (*p* < 0.01). In addition, the alteration of pro- and anti-inflammatory cytokine levels, such as IL-6, IL-1β, TNF-α, and IL-10, in the serum of the DSS-induced colitis mice were determined by ELISA. From the Figure 3B, the significantly higher concentrations of IL-6, IL-1β, and TNF-α and markedly lower levels of IL-10 in DSS mice were observed compared with NC mice. And treated with algal oil at high dosage decreased the levels of IL-6, IL-1β, and TNF-α, and elevated the level of IL-10 (*p* < 0.05).

### Algal Oil Administration Protected the Intestinal Barrier in Colitis Mice

**FIGURE 4:**
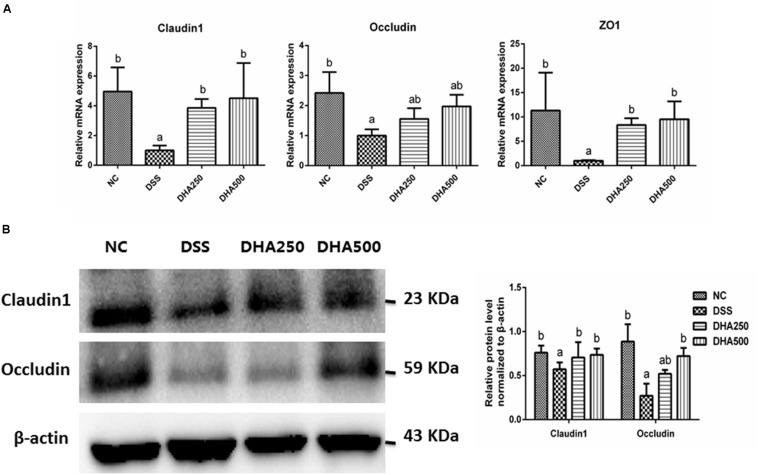
Effects of algal oil on the intestinal barrier of mice with colitis induced by DSS. **(A)** the mRNA level of Claudin-1, Occludin, and ZO-1, **(B)** the expression of Claudin-1, Occludin proteins. The results are expressed as the mean ± SEM (*n* = 9). Groups with the same letter are considered to have no significant differences, and groups with different letter are significantly different at *p* < 0.05. Among them, the average data of group with “a” was the minimum while the average data of group with “b” was the maximum.

To assess the effects of algal oil on the intestinal barrier in colitis mice, the mRNA levels of Claudin-1, Occludin, and ZO-1 and the protein levels of Claudin-1 and Occludin in colon tissues were determined. As Figure 4A shown, the mRNA levels of Claudin-1, Occludin, and ZO-1 were significantly decreased in the mice with colitis when compared with that in the NC group (*p* < 0.05). However, administration of algal oil significantly increased the mRNA expression levels of Claudin-1, Occludin, and ZO-1 in the DSS-treated mice. Additionally, the Claudin-1 and Occludin protein expression levels were also found to significantly decrease in mice from the DSS group, while algal oil treatment, especially 500 mg/kg/day of algal oil, obviously reversed the changes of Claudin-1 (*p* < 0.05) and Occludin (*p* < 0.05) expression in colitis mice (Figure 4B).

### Algal Oil Regulated Structure and Composition of Gut Microbiota in Mice

**FIGURE 5:**
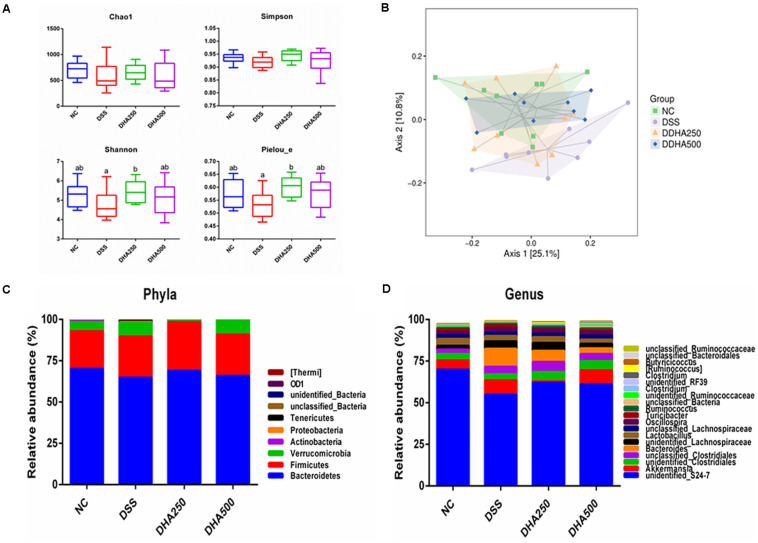
Analysis of the differential microbial community among the groups. **(A)** Alpha diversity indexes (Chao1, Shannon, Simpson, and Pielou_e) of the gut microbiota, **(B)** PCoA diagram based on the unweighted UniFrac distances of beta diversity of the gut microbiota, **(C)** gut microbial compositions at phylum level (Only phyla with top 10 average abundances were included), and **(D)** gut microbial compositions at genus level (Only phyla with top 20 average abundances were presented.) The results are expressed as the mean ± SEM (*n* = 9). Groups with the same letter are considered to have no significant differences, and groups with different letter are significantly different at *p* < 0.05. Among them, the average data of group with “a” was the minimum while the average data of group with “b” was the maximum.

The structure and composition of the gut microbiota in algal oil-treated mice were analyzed using the 16S rDNA sequencing technology. In the alpha diversity index of each sample, Chao1 mainly represents the sample community richness and Simpson, Shannon and Pielou_e indices mainly reflect the sample species diversity. Figure 5A showed that the Shannon (*p* < 0.05) and Pielou_e indices (*p* < 0.05) were higher in the DHA250 group than in the DSS group. There was no statistical significance in the Chao1 index and Simpson index among each group. As shown in Figure 5B, the microbial structure of mice in the NC and DSS groups were remarkable different, and administration of algal oil could remit the shift of gut microbiota structure induced by DSS treatment.

**FIGURE 6:**
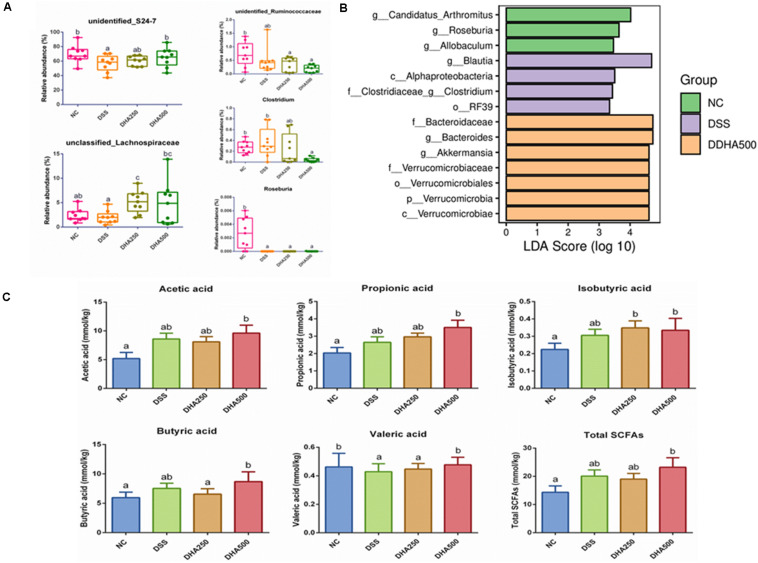
Composition analysis of intestinal microbiota and change of SCFAs contents in all groups. **(A)** The relative abundance of representative bacteria at genus level, **(B)** LDA-LEfSe analysis, **(C)** concentrations of SCFA of caecum contents in each group. The results are expressed as the mean ± SEM (*n* = 9). Groups with the same letter are considered to have no significant differences, and groups with different letter are significantly different at *p* < 0.05. Among them, the average data of group with “a” was the minimum while the average data of group with “b” was the maximum.

The composition of gut microbiota of each group of mice at the phylum and genus levels was shown in Figures 5C,D. At the phylum level, the gut microbiota of each sample was mainly consisted of *Bacteroidetes*, *Firmicutes*, *Verrucomicrobia* and other phyla. At the genus level, the main bacterial genera detected in each group were *unidentified_S24_7*, *Akkermansia*, *unidentified_Clostridiales*, *unclassified_ Clostridiales*, and *Bacteroides*. In addition, from the Figure 6A, we found DSS treatment significantly decreased the proportion of *unidentified_S24_7* (*p* < 0.05) when compared with NC group, while supplementation with algal oil reversed the relative abundance of this genus in colitis mice. Algal oil administration significantly increased the relative abundance of *unclassified_Lachnospiraceae* when compared to NC or DSS groups. The relative abundance of *unidentified_Ruminococcaceae*, *Clostridium*, and *Roseburia* in DHA groups was less than those in NC and DSS groups.

The prominent taxa in each group were also analyzed based on LDA-LEfSe, which revealed that *Candidatus_Arthromitus* and *Roseburia* were enriched in the NC group, whereas, *Blautia* and *Alphaproteobacteria* were enriched in the DSS group, *Bacteroidaceae* and *Bacteroides* were enriched in the DHA500 group and there was no specific biomarker found in DHA250 group (Figure 6B).

### Algal Oil Altered the Caecal SCFAs Profile

The bacterial metabolite SCFAs in caecal contents were determined by GC and SCFAs profile in each group was presented in Figure 6C. It has been found that there were no remarkable differences between NC group and DSS group in the contents of acetic acid, propionic acid, isobutyric acid, buturic acid and the total SCFAs. And the contents of acetic acid (*p* < 0.05), propionic acid, isobutyric acid (*p* < 0.05), buturic acid (*p* < 0.05), valeric acid (*p* < 0.05) and the total SCFAs (*p* < 0.05) in the DHA500 group were higher than that in the DSS group. Algal oil treatment at the dose of 500 mg/kg/day dramatically increased the content of valeric acid to normal level compared with the DSS group (*p* < 0.05). No differences were observed in the production of isovaleric acid among all groups (not shown).

### Correlation Between the Indexes About Oxidative Stress and Cytokines With Gut Microbiota

**FIGURE 7:**
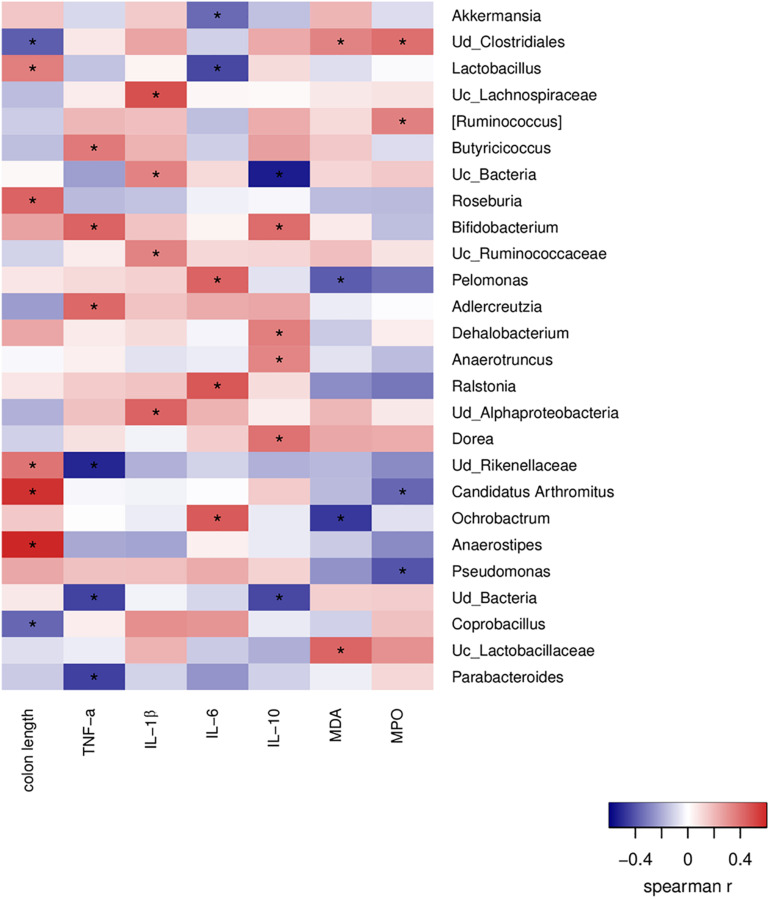
Correlations of representative microbial taxa, oxidative stress markers and inflammatory cytokines among each group. Spearman’s rank correlation coefficients were calculated. Red indicates positive correlations, and blue indicates negative correlations. Significant differences were indicated as *P < 0.05.

A correlation analysis between the altered intestinal bacteria, inflammatory cytokines and oxidative stress markers was performed to further explore the protective effects of algal oil-modified gut microbiota. It has been found that 26 genera were closely correlated with at least one colitis-related index negatively or positively (Figure 7). The colon length was positively correlated with the populations of *Roseburia*, *Candidatus Arthromitus*, and *Anaerostipes*, but negatively correlated with *unidentified_Clostridiales* and *Coprobacillus*. Altered genus of *unclassified_ Bacteria*, *unidentified_Alphaproteobacteria* and *Ralstonia* were positively related with pro-inflammation cytokines secretion (TNF-α, IL-1β, and IL-6), but were negatively related with IL-10. In addition, the markers of oxidative stress showed positive correlations significantly with *unidentified_Clostridiales* and *unclassified_Lactobacillaceae*, whereas *Pelomonas* and *Ochrobactrum* negatively correlated with the levels of MDA and MPO.

## Discussion

In the current study, we studied the therapeutic effects of algal oil rich in DHA and DPA on colitis induced by DSS and tried to explore its potential mechanism. Two fatty acids have been confirmed to be beneficial for body’s immune reaction and have been considered to be the most representative components for algal oil according to nutrition characteristics and bioavailability studies of algal oil (Barroso-Hernández et al., 2020). DHA administration has been reported to inhibit the production of cytokine IL-1β related to inflammation in mice (Rong-Hui et al., 2018). Furthermore, one study showed that DHA exerted anti-inflammatory effect in the aspect of suppressing pulmonary metastases and proliferation of melanoma cells. In addition, DPA exerted inhibitory effects on intracellular levels of cyclooxygenase-2 and pro-inflammatory prostaglandin E2 and pro-inflammatory cytokines production (Nauroth et al., 2010). Thus, it would be assumed that the anti-inflammatory effect of algal oil might be related to the presence of DHA and DPA. However, further research may be needed to better understand the observed effects.

We demonstrated that treatment with algal oil alleviated DSS-induced a series of colitis symptoms. In this study, the evaluation of colitis was based on the macroscopic and microscopic tissue injury, and supplementation with algal oil was found to significantly relieve colon injury. Moreover, DSS-induced colitis caused an increase of the MPO and MDA production in colon tissues, and these phenomenons were significantly reversed by algal oil treatment, which was in line with the reduction in neutrophil cell infiltration into colonic mucosa observed through histopathological analysis.

IBD is featured by dysregulated immune function, imbalanced cytokine release and intricate inflammatory progress related to intestinal mucosa (Libby et al., 2012). Patients suffering from IBD have high levels of pro-inflammatory cytokines in the gut mucosa, such as IL-1β, IL-6, and TNF-α (Morshedzadeh et al., 2019). These cytokines are involved in the disruption of TJs in the epithelial layer through the nuclear factor-κB signaling pathway (Liu et al., 2013). Furthermore, TNF-α and IL-6 could mediate monocyte cell infiltration into the intestinal tissues to result in tissue injury (Liu et al., 2014). Thus, inhibition the production and release of these inflammatory factors may offer an important strategy for IBD therapy. In our study, we found the increased productions of IL-1β, IL-6, and TNF-α stimulated by DSS treatment were significantly suppressed by algal oil, while the decreased level of IL-10 caused by DSS was reversed by algal oil. The results revealed that the ameliorative effect of algal oil against DSS-induced colitis in mice might be closely related to its inhibition on pro-inflammatory cytokines productions and promotion on anti-inflammatory cytokines release.

The intestinal epithelial cells linked together by TJ proteins prevent the free crossing of small molecules, ions and bacteria through the space between adjacent epithelial cells (Ahmad et al., 2017). Maintaining the normal expression and function of TJ proteins and reducing intestinal epithelial barrier permeability were critical for IBD therapy. Claudin-1 protein deleted mice were at high risk of dying for dehydration, which suggested that this protein played an important role in maintaining the barrier function (Furuse et al., 2002). Occludin, a key member of transmembrane protein, mainly regulated the permeability of TJs and maintained epithelial cell polarity. Zos which belong to a kind of peripheral membrane proteins, had the ability to connect Claudins, Occludin, and other cytoskeletal proteins to maintain the integrity of TJs complex (Nunes et al., 2019). Consistent with the previous study (Li et al., 2020), treatment with DSS resulted in the decrease of mRNA and protein expression of Claudin-1, Occludin, and ZO-1. Algal oil administration improved the expression of Claudin-1, Occludin, and ZO-1 in colonic tissues. Therefore, algal oil supplementation was an effective treatment option on making the intestinal barrier function in colitis mice normal.

Gut microbiota disorder is a critical factor in the process of IBD. Different types of bacteria play different roles in inflammatory process, and some intestinal microbiota are beneficial for inflammatory response, while some can douse inflammation (Frank et al., 2007). Several changes in the gut microbiota of IBD patients occur, and individuals with IBD showed a low microbiota diversity (Manichanh et al., 2006). It has been found that groups between NC and DSS did not show significant difference in the aspect of microbiota diversity in this study, while the diversity of gut microbiota had improved somewhat after algal oil treatment. The results of PCoA showed that DSS treatment altered the structure of gut microbiota, while algal oil showed a positive regulatory role on the gut microbiota community in the DSS-induced colitis mice. The salient feature of algal oil treatment group was remarkably increased in the numbers of *unidentified_S24-7* genus. It has been proved that *unidentified_S24-7* can contribute to the release of extracellular DNA in mucus layer of colon tissue, which caused lower levels of IL-6, TNF-α, and higher level of IL-10 to maintain small intestinal immune homeostasis (Qi et al., 2017). Wu et al. (2020) also demonstrated the beneficial effect of *unidentified_S24-7* on alleviating DSS-induced colitis (Xinyue et al., 2018). As a SCFA-producer, the population of genus *unclassified_Lachnospiraceae* was negatively related to inflammation (Rong-Hui et al., 2018). Consistently, our study also found that the proportion of *unclassified_Lachnospiraceae* was reversed by algal oil treatment in colitis mice. It seemed that the protective effect of algal oil was attributed to its regulatory effect on beneficial bacteria, which was correlated with immune responses and their metabolites.

Compositional alterations of the gut microbiota may also influence the metabolic capacity of the gut bacteria (De Preter et al., 2015). SCFAs, the major fermentation products of dietary fiber in gut, have been proved to be critical for maintaining intestinal homeostasis and relieving colitis through strengthening the intestinal barrier function and immunomodulatory function (Parada Venegas et al., 2019). In particular, butyric acid is the main energy source for colonic epithelial cells, and acetic acid participated in the production of fat and gluconeogenesis process (Shao et al., 2019). Importantly, IBD patients showed decreased abundance of SCFAs-producing bacteria (like *Lachnospiraceae* and *Roseburia*) in intestinal mucosa and caecal contents, and the levels of SCFAs appeared to be lower when compared to healthy individuals (Wang et al., 2014). In this study, there was a closely relation between regulated intestinal bacteria and the content of SCFAs. Algal oil treatment stimulated the production of acetic acid, propionic acid, isobutyric acid, butyric acid, and valeric acid, which was consistent with the significant increase of the SCFAs-producing bacteria, such as *unclassified_Lachnospiraceae*. As the main butyrate producing-bacteria, the increased proportion of *Lachnospiraceae* can promote the level of butyrate in the intestines, further playing a positive role in maintaining intestinal homeostasis by supporting epithelial cell proliferation and promoting the epithelial barrier function (Louis and Flint, 2009, 2017). Thus, the alleviation of DSS-induced colitis by algal oil treatment might also rely to the regulation of the gut bacteria metabolism.

Recent researches suggest that modulation of gut microbiota alleviated colitis through the direct regulation of the host immune system and the enrichment of metabolite SCFAs, which could inhibit proliferation of epithelial cells and regulate the inflammatory responses (Kang and Martin, 2017; Zeng et al., 2019). This article mainly focuses on the effect of algal oil rich in DHA on colitis in mice, and the potential underlying mechanisms by which algal oil alleviate inflammation still needs further study. In this study, we found that algal oil treatment altered the structure of gut microbiota and increased the proportion of beneficial bacteria, like *unidentified_S24-7* and *unclassified_Lachnospiraceae* genera. Significantly, *unidentified_S24-7* contributes to maintain the balance of cytokines and small intestinal immune homeostasis, and *unclassified_Lachnospiraceae* helps the production of butyrate in the gut. So we speculated that the protective effect of algal oil was attributed to its regulatory effect on beneficial bacteria. However, more in-depth studies and investigations are needed to verify this hypothesis.

## Conclusion

Our study confirmed that supplementation of algal oil alleviated the intestinal inflammation and exerted a protective effect on maintaining intestinal epithelial barrier function in colitis mice. Algal oil treatment restored various biophysical variables, including the increase of body weight and colon length and the decrease of DAI score. The concentration of pro-inflammatory cytokines was decreased by the supplementation of algal oil. Algal oil could increase TJ proteins expression, maintain intestinal integrity and improve intestinal epithelial barrier function. Moreover, algal oil played a positive role in regulating the intestinal microbial composition and structure and promoted the production of SCFAs. These results will contribute to understand the functions of algal oil in alleviating intestinal inflammation and regulating immune-mediated diseases, thus guide further development and usage of dietary lipid products.

## Data Availability Statement

The datasets presented in this study can be found in online repositories. The names of the repository/repositories and accession number(s) can be found below: https://www.ncbi.nlm.nih.gov/, PRJNA668242.

## Ethics Statement

The animal study was reviewed and approved by the Experimental Animal Center of Huazhong Agricultural University under the approved protocol number SYXK 2015-0084.

## Author Contributions

ZX and CY designed the experiments. ZX conducted the experiments, analyzed the data, and wrote the manuscript. CY, HT, and ZQ revised the manuscript. XW performed project administration of the animal experiment. CY, FH, and QD acquired the funding and supervised the project. All authors agreed to be accountable for all aspects of the work.

## Conflict of Interest

The authors declare that the research was conducted in the absence of any commercial or financial relationships that could be construed as a potential conflict of interest.

## Supplementary Material

Supplementary Table 1 The fatty acid composition and the relative content of algal oil.

Supplementary Table 2 The composition of the standard diet used in this study.

Supplementary Table 3 Primer sequences used in RT-qPCR assays in colonic tissue.
