# Supplementary material for: Algal Oil Rich in n-3 PUFA Alleviates DSS-Induced Colitis via Regulation of Gut Microbiota and Restoration of Intestinal Barrier
Source: Front Microbiol. 2020 Dec 16;11:615404. doi: 10.3389/fmicb.2020.615404 (PMC7772400; doi:10.3389/fmicb.2020.615404)
Supplement: Supplementary Table 1 — The fatty acid composition and the relative content of algal oil. [file Data_Sheet_1.docx]

Supplementary Material

## Supplementary Tables

**Table S1.** The fatty acid composition and the relative content (%) of DHA algal oil.

| C14:0 | C15:0 | C16:0 | C16:1 | C17:0 | C17:1 | C18:0 | C18:1 | C18:2 | C18:3 | C20:4 | C20:5 | C22:5 | C22:6 |
| --- | --- | --- | --- | --- | --- | --- | --- | --- | --- | --- | --- | --- | --- |
| 3.78±0.05 | 0.56±0.01 | 20.44±0.16 | 0.83±0.004 | 0.36±0.01 | 0.37±0.002 | 1.02±0.004 | 5.05±0.03 | 1.32±0.02 | 0.37±0.003 | 1.59±0.003 | 1.10±0.03 | 16.52±0.06 | 46.70±0.17 |

Results are expressed as the mean ± SEM.

**Table S2.** The composition of a standard diet.

| AIN93M | gm% | kcal% |
| --- | --- | --- |
| Fat | 4 | 9.4 |
| Protein | 14.2 | 14.7 |
| Carbohydrate | 73.1 | 75.9 |
| kcal/gm |  | 3.85 |
| Ingredient | gm | kcal |
| Casein, 30 Mesh | 140 | 560 |
| L-Cystine | 1.8 | 7.2 |
| Corn Starch | 495.692 | 1983 |
| Maltodextrin 10 | 125 | 500 |
| Sucrose | 100 | 400 |
| Cellulose | 50 | 0 |
| Soybean Oil | 40 | 360 |
| t-Butylhydroquinone | 0.008 | 0 |
| Mineral Mix S10022M | 35 | 0 |
| Vitamin Mix V10037 | 10 | 40 |
| Choline Bitartrate | 2.5 | 0 |
| Total | 1000 | 3850 |
| *Soluble fiber/carbohydrate | 540.85 |  |
| *Insoluble fiber/carbohydrate | 50.49 |  |

The total carbohydrate of corn starch was calculated as 85% with 0.1% insoluble fiber, and the total carbohydrate of maltodextrin was calculated as 96%. The content of soluble and insoluble fiber in the diet slightly varies on the different batch of the corn starch and maltodextrin.

**Table S3.** Primer sequences used in RT-qPCR assays in colonic tissue.

| Targer gene | Sequence (5’-3’) | NCBI gene ID |
| --- | --- | --- |
| Claudin 1 | F: AGCTGCCTGTTCCATGTACT | ID: 12737 |
|  | R: CTCCCATTTGTCTGCTGCTC |  |
| Occludin | F: AGCACTTAACCTGCCTGGAT | ID: 18260 |
|  | R: AGCCTGTGGAAGCAAGAGAT |  |
| ZO 1 | F: TGAGTGCGTTTCTCTCCCTT | ID: 21872 |
|  | R: CCCTCTGTGTTCCTCATGGT |  |
| GAPDH | F: GGACTTACAGAGGTCCGCTT | ID :14433 |
|  | R: CTATAGGGCCTGGGTCAGTG |  |
