# Supplementary figures and images for: Algal Oil Rich in n-3 PUFA Alleviates DSS-Induced Colitis via Regulation of Gut Microbiota and Restoration of Intestinal Barrier
Source: Front Microbiol. 2020 Dec 16;11:615404. doi: 10.3389/fmicb.2020.615404 (PMC7772400; doi:10.3389/fmicb.2020.615404)

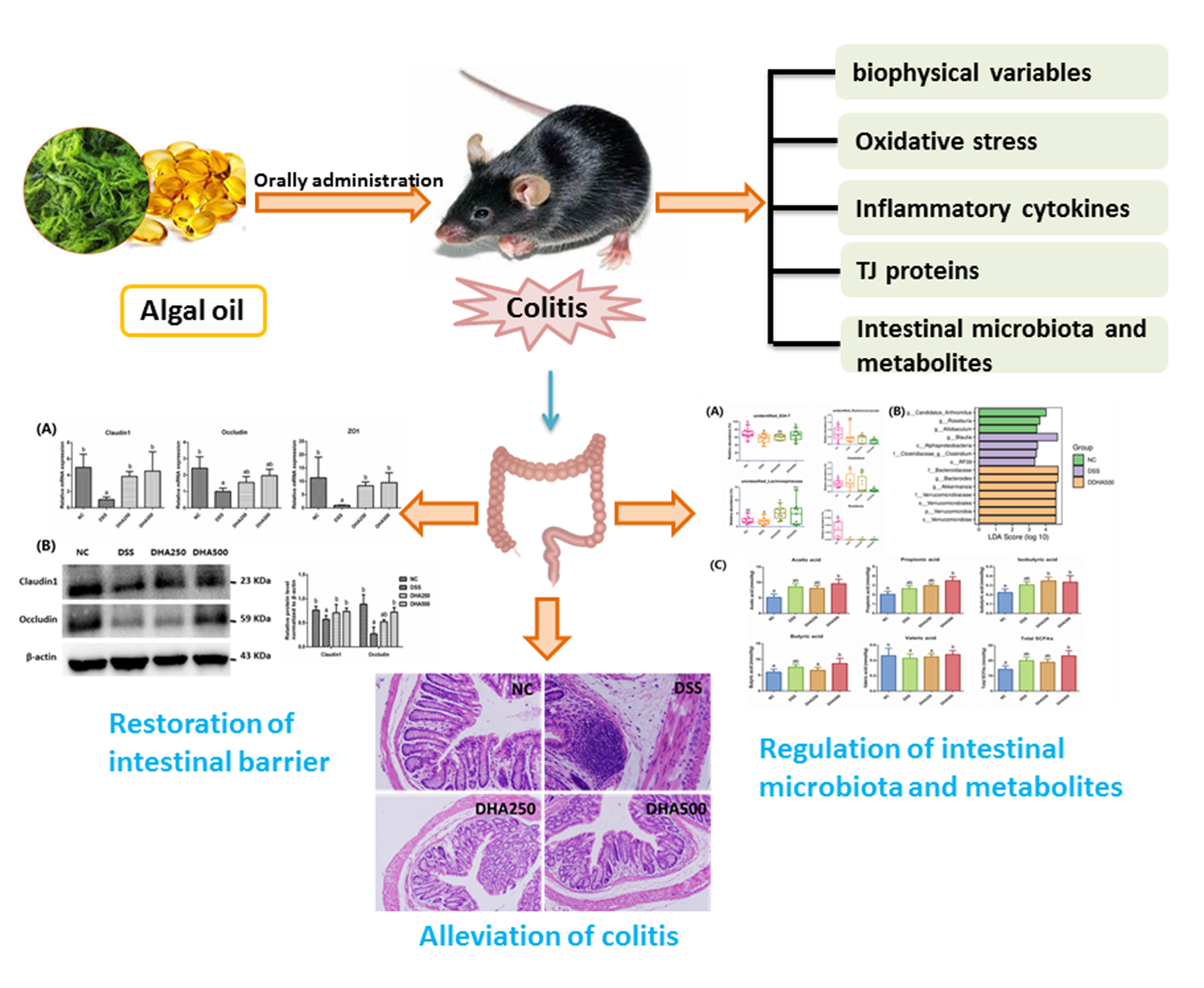

Supplement: Supplementary file 2 [file Image_1.TIF]

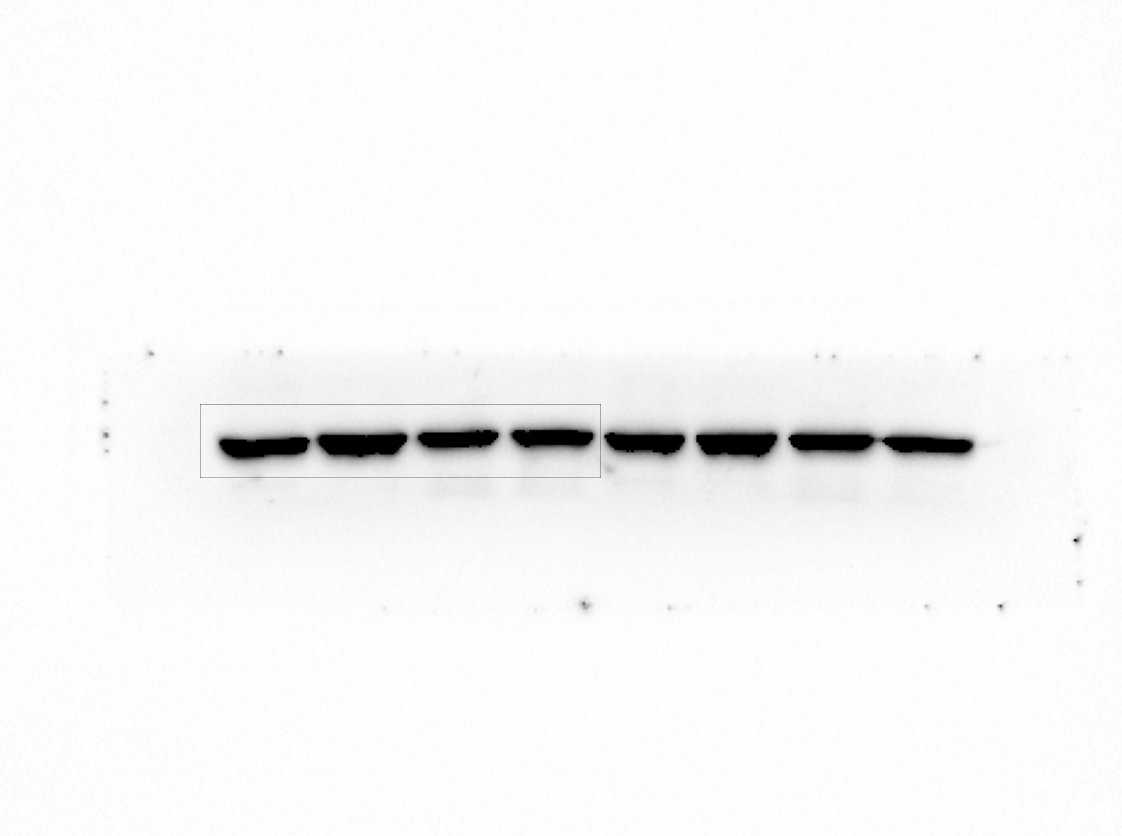

Supplement: Supplementary file 3 [file Image_2.TIF]

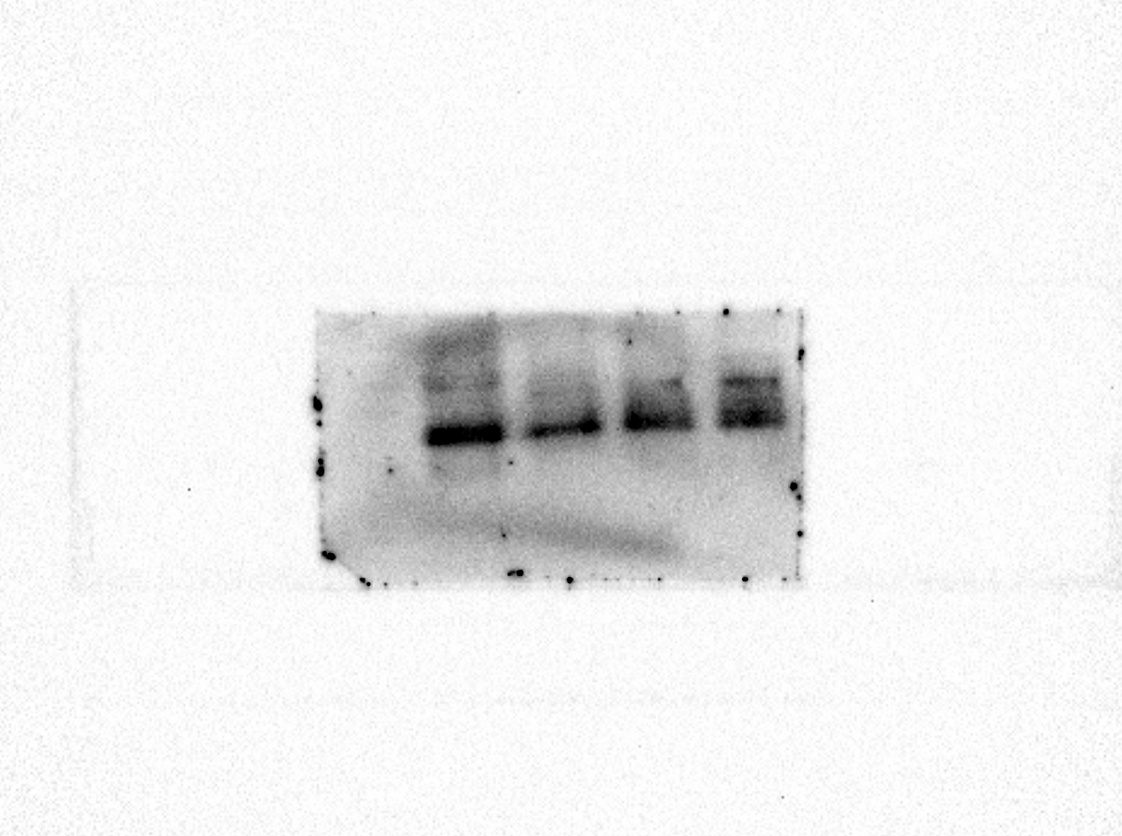

Supplement: Supplementary file 4 [file Image_3.TIF]

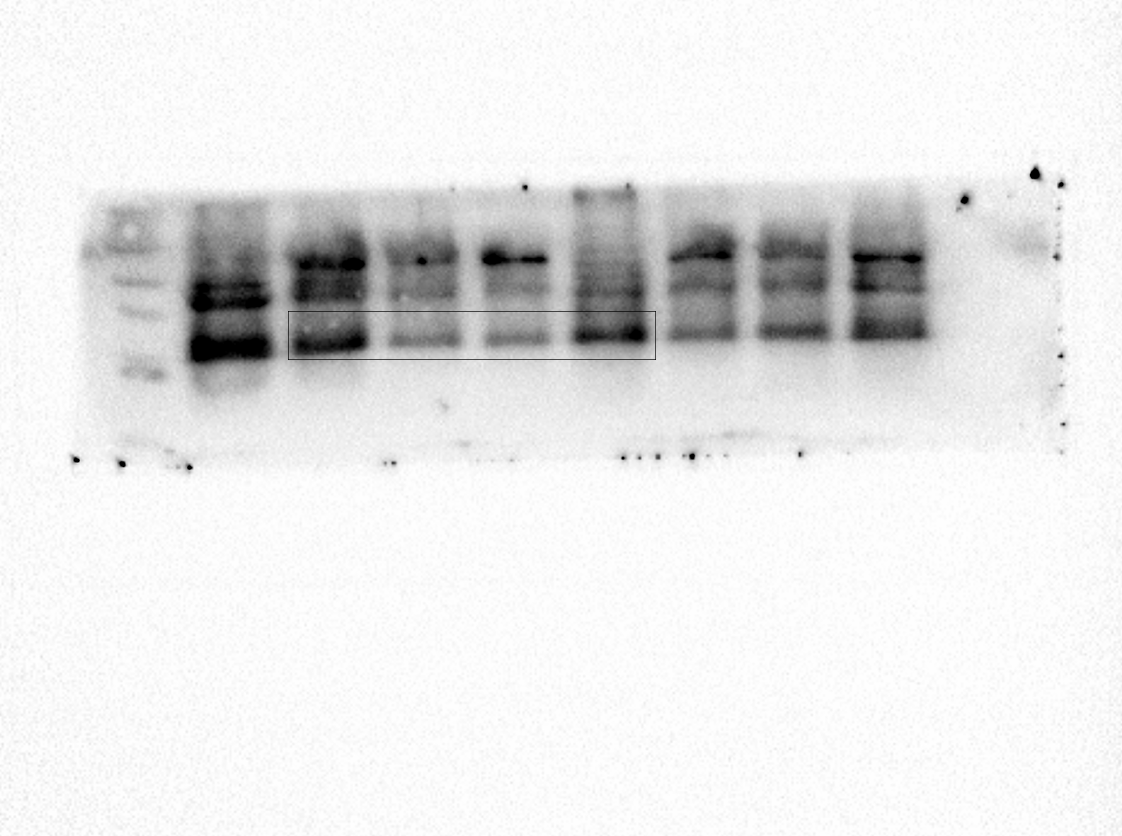

Supplement: Supplementary file 5 [file Image_4.TIF]
